# MaizeCUBIC: a comprehensive variation database for a maize synthetic population

**DOI:** 10.1093/database/baaa044

**Published:** 2020-06-16

**Authors:** Jingyun Luo, Chengcheng Wei, Haijun Liu, Shikun Cheng, Yingjie Xiao, Xiaqing Wang, Jianbing Yan, Jianxiao Liu

**Affiliations:** 1 National Key Laboratory of Crop Genetic Improvement, Huazhong Agricultural University, Wuhan 430070, China; 2 College of Informatics, Huazhong Agricultural University, Wuhan 430070, China; 3 Gregor Mendel Institute, Austrian Academy of Sciences, Vienna Biocenter, Vienna 1030, Austria

## Abstract

MaizeCUBIC is a free database that describes genomic variations, gene expression, phenotypes and quantitative trait locus (QTLs) for a maize CUBIC population (24 founders and 1404 inbred offspring). The database not only includes information for over 14M single nucleotide polymorphism (SNPs) and 43K indels previously identified but also contains 660K structure variations (SVs) and 600M novel sequences newly identified in the present study, which represents a comprehensive high-density variant map for a diverse population. Based on these genomic variations, the database would demonstrate the mosaic structure for each progeny, reflecting a high-resolution reshuffle across parental genomes. A total of 23 agronomic traits measured on parents and progeny in five locations, where are representative of the maize main growing regions in China, were also included in the database. To further explore the genotype–phenotype relationships, two different methods of genome-wide association studies (GWAS) were employed for dissecting the genetic architecture of 23 agronomic traits. Additionally, the Basic Local Alignment Search Tool and primer design tools are developed to promote follow-up analysis and experimental verification. All the original data and corresponding analytical results can be accessed through user-friendly online queries and web interface dynamic visualization, as well as downloadable files. These data and tools provide valuable resources on genetic and genomic studies of maize and other crops.

## Introduction

Maize (*Zea mays*) is one of the most diverse crops containing tremendous variation, making it a perfect model for both genetic and genomic studies in plants. Next-generation sequencing technology (NGS) is a powerful and cost-effective approach for discovering genetic variation and rapidly becoming a desirable choice for population-level genomic studies. The first-generation haplotype map of maize was constructed in 2009, millions of SNP obtained by low-coverage sequencing among 27 diverse maize inbred lines (7). Since then, more maize lines with higher sequencing depth were applied to develop the maize haplotype map. The maize HapMap2 was constructed with the whole-genome sequencing data of 103 lines across pre-domestication and domesticated *Z. mays* varieties, identified over 55 million SNPs (5). And the latest released maize haplotype version 3 built from whole-genome sequencing data among 1218 collected maize lines, identified more than 80 million variants (4). These increasing genomics data sets provide valuable resources for the maize community. To make better use of these genomic resources, convenient and professional database platforms are required to be constructed.

With the rise of computer information and networking technologies, several databases for maize genomics and functional genomics have been developed, including MaizeGDB (21), which collects several released maize genomes, diverse germplasm, phenotypic and genotypic data and also provides some effective analytical tools. It is one of the most commonly used and well-known maize database. Panzea (27), for example, contains genotypic and phenotypic data of several maize populations. MODEM (http://modem.hzau.edu.cn/) integrates multi-omics data sets of 527 maize elite inbred lines, including genomic, transcriptomic, metabolic and phenotypic information (17). Other generic databases, such as GenBank (3), Gramene (23) and ePlant (25) also contain maize omics data. These collections of high-quality genomic resources and various database platforms provide valuable resources for maize research and breeding.

However, the analytical tools developed for the population genomics research in current databases are left behind, especially the lack of tools for visualization of genetic mapping results and further follow-up analyses. Moreover, most databases mentioned above are designed to natural un-related panels, in which individuals with unknown kinship are selected. Such populations always have obvious population structure and low-frequency functional alleles, which limit its efficacy in the genetic mapping of complex agronomic traits. Geneticists and breeders attempt to use diverse genetic mating designs in a controllable hybridization to develop large-scale synthetic populations that are more efficient for genetic mapping. RIL populations derived from bi-parental crosses, such as the B73 x Mo17 (IBM) population (12), are traditional mating designs in plant genetic research, however, suffer from insufficient allelic diversity and recombination events. To address these problems, the creation of inbred lines derived from multi-parent cross designs has been used, such as the nested association mapping (NAM) panel (20) and the multiple-parent advanced generation inter-cross (MAGIC) population (6). Maize NAM population was developed using 25 maize inbred lines crossed to a common recurrent parent (B73) to develop around 200 lines for each of 25 RIL populations, resulting in a total of 5000 recombinant inbred lines (26); however, the incapable of crossing among the 25 founders would potentially reduce the diversity of haplotype combinations. And the B73 parent alleles being sampled many more times than those of other founders, which is less statistically efficient and limits mapping power (8). The MAGIC population overcomes this shortcoming by pooling two-way, four-way and eight-way hybrids in independent breeding funnels, but the number of founders in MAGIC panel typically requires to be fixed (2^N^, N is a positive integer usually greater than 2) and the development cycle is usually long (N generations are needed to mix all parents, N is the index of 2 mentioned above).

**Table 1:** Resources in MaizeCUBIC database

Index	Data description
Population introduction	The present CUBIC population, consisting of 1404 progenies, was derived from 24 elite Chinese maize inbred lines from four divergent heterotic groups.
Genomic variation	Over 14M SNPs, 43K InDels. 660K SVs, 600M novel sequences.
Phenotype data	A total of 23 agronomic traits were measured on parents and progenies in five locations representative of where maize is mainly grown in China.
Expression data	A subset of 391 progenies was randomly selected from the CUBIC population for RNA-sequencing, and the gene expression quantifications data and eQTL mapping results was collected.
Haplotype bin map	The mosaic structure for 1404 progenies in population was successfully reconstructed.
QTL mapping	Mapping results of association analysis for traits by two different methods.

**Figure 1:**
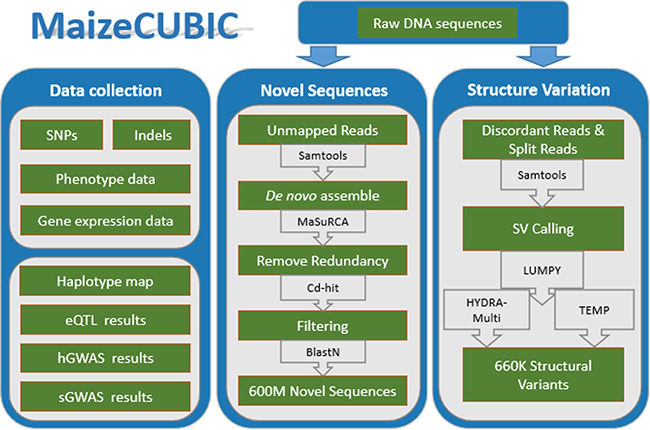
The MaizeCUBIC data integration and processing workflow.

Compared to them, data in MaizeCUBIC were collected from a well-designed large maize population named complete-diallel plus unbalanced breeding-derived inter-cross (CUBIC). A total of 1404 offspring inbreds are derived from two rounds of complete-diallel design among 24 founders, following with six generations of open pollination and another six generations of selfing (16). CUBIC population combines the advantages of both the diallel cross and MAGIC designs, with its high genetic diversity, large population size, sufficient and traceable recombination events and reduced development period. These together make it more promising for genomic and genetic analysis. The population has been sequenced at low coverage, following with conventional variant calling and imputation processes, and over 14M SNPs and 43K indels were obtained (16). Beyond these reference-based small genomic variations, additional 660K SVs and 600M novel (non-B73 reference) sequences were newly identified in this study, representing a comprehensive high-density variant map for the CUBIC population. Based on these genomic variations, each descendant inbred line is clearly traced as a mosaic structure map describing high-resolution recombination among different founders. This detailed haplotype map makes it possible to study how short term mutation, recombination and selection reshape such dramatic variations between descendants, providing new insights for maize breeding. A total of 23 traits measured on parents and progeny in five locations were also available in this database. To further explore the genotype–phenotype relationships, two different methods of GWAS were provided for dissecting genetic mechanisms of those agronomic traits. MaizeCUBIC also developed useful tools for users to easily search, analyze and visualize all these different variation data and corresponding analytical results. The resource list in MaizeCUBIC database is shown in Table 1. Altogether, this database would provide wide-ranging benefits for researchers in many fields.

## Materials and Methods

### Database implementation

MaizeCUBIC is developed using the framework Struts 2 (9), boostrap, jQuery, bokeh, echart and B/S development pattern. The back end of MaizeCUBIC is implemented in Java, and the web interface is implemented using JSP, JavaScript, HTML5 Canvas and AJAX technologies. The webpage access service is provided using the Apache Tomcat as the server and the MySQL database as the management system. These technologies allow the user to search and display their assignments conveniently and efficiently. All scripts involved in this study have been deposited into Github (https://github.com/Tfrain/Cubic). The data in the MaizeCUBIC database contains published variations of the CUBIC population and newly identified structure variations (SVs) and novel sequences of the population, the integrated processes are roughly shown in Figure 1.

### Data collection

The CUBIC population consists of 1404 progenies descended from 24 Chinese elite inbred lines (Supplementary Table 1). The genotype and phenotype data were collected from a previous study (16). Briefly, over 14.12 million SNPs and 43K indels were obtained, and the SNP set was found ~97% consistency compared with genotypes derived from array- and assembly-based methods before. The population was planted in five different provinces and a total of 23 agronomic traits were investigated (see Phenotype Data introduction page for the detailed list). All these genomic variation data and phenotype data can be accessed through the analytical tools, as well as downloaded directly from our links or File Transfer Protocol site. Also, based on these variations, the mosaic haplotype map for progenies in the population and the QTL mapping results of two different GWAS methods was provided through user-friendly online queries and web interface dynamic visualization tools.

### Identification of SV

Clean reads were mapped to the B73 reference genome (v3.25, downloaded from http://plants.ensembl.org) using BWA-MEM (version 0.7.12, 14) and discordant reads (with unusual insert size) and split ones (best mapped to clipped positions) were extracted and applied in SV calling (as population mapping-based strategy). The software LUMPY (11) and HYDRA-Multi (15) were integrated in the SV calling. Another assembly-based SV calling strategy was performed through comparing the B73 reference genome into HuangZaoSi (HZS) scaffolds (13), for which the MUMmer (release 3.0,) (10) was implemented to achieve.

### Novel sequences assemble

Reads that could not be aligned to the B73 reference genome (v3.25), including unmapped reads from both parents and progeny, were used to generate novel non-B73 sequences. Due to the large number of unmapped reads, we divided these sequences into several batches when using MaSuRCA (v3.13,) (28) software for the *de novo* assembly. The CD-HIT (v4.6.5) (18) software with default parameters was next applied to clustering all the assembled contigs to remove redundancy sequences. Then these non-redundant contig sets were aligned to the B73 reference genome (v3.25), and those alignments with concordance > = 90% and coverage > = 50% was removed from future analysis. Additionally, these novel sequences were filtered against The European Bioinformatics Institute bacterial genome database (EBI; http://www.ebi.ac.uk) and B73 plastid genome sequences (v3.25) using basic local alignment search tool (BLAST) (2) requiring a minimum e-value of 1e-5, a minimum of 50% coverage and 85% identity. Also, any sequences tend to be non-Plantae was removed from downstream analyses. Finally, about 600M novel sequences were obtained, including 464 707 contigs. To evaluate the reliability of novel sequences, we compared them with the PAV tags in a study of maize pan-genome sequence anchors (19), 148 916 (32%) of our novel sequences was aligned to 818 056 (71.3%) of the PAV tags with a minimum of 90% coverage and 85% identity. Also, the novel sequences were BLAST to a HZS genome (13), nearly 17% (79 000 of 464 707) of novel sequences had above 85% identity. In brief, these novel sequences are reliable and cover wider variation than previous studies.

## Results

### Database features

The tools in our database can be grouped into three general classes according to its related information: variation map (haplotype bin map, genome browser, variation search, gene expression search), GWAS catalog (GWAS diagram, GWAS search) and variation application (BLAST/Primer-BLAST, general primer design, primer design by region, primer design by variation ID).

### Haplotype bin map

**Figure 2:**
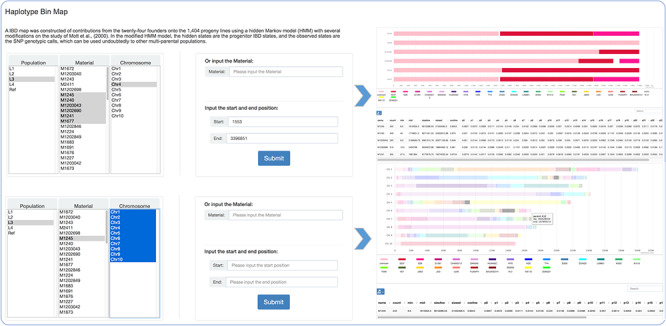
Haplotype bin map tools developed in MaizeCUBIC to display the mosaic structure for progenies in population.

The mosaic structure for progenies in population was successfully reconstructed using a modified hidden Markov model (16). It is a clear reflection of recombination and parent contribution in population, which is very helpful in the next step genetic analysis. The population option refers to divergent subpopulations shaped by short-term artificial selection to maintain the maximum of phenotypic diversity. Within the principal components analysis and admixture (v1.3.0) (1) analyses, L1–L4 were revealed to indicate four small sub-groups within the offspring population, consisting of 54 to 70 individuals each. The remainder formed one large group that was seemingly to be randomly derived from the 24 founders, which was regarded as Ref (the reference panel). Users can search for detailed haplotype information through choosing or entering accession names and selecting the corresponding genomic region (Figure 2). Focusing on details of one material and comparison analysis of multiple materials are both feasible.

### Genome browser

**Figure 3:**
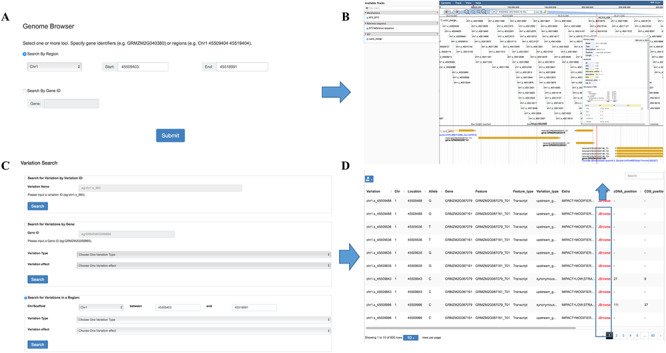
Features of MaizeCUBIC variation module. (**A**) Variation information of population can be visual browsed by searching for chromosome region or gene names in genome browser tool. (**B**) Schematic of genome browser embedded in MaizeCUBIC, build on JBrowse. (**C**) Variations can be queried in three ways in variation search tool. (**D**) The acquired variations would be displayed in the results page within a table and have links to the relevant entry in genome browser tool to see detail information about them.

Genome browser is a utility enabling researchers to easily browse sequences, genes and genetic variations in various regions. It was built on one of the most widely used genome visualization tools JBrowse (22), a JavaScript-based genome browser with a fully dynamic AJAX interface, which is very fast and scales well to large data sets. Variation information among the population can be browsed by searching for chromosome region or gene names in this tool (Figure 3A). Several tracks are available allowing a user to retrieve multiple kinds of data simultaneously and to analyze the integrated data (Figure 3B). The ‘Reference sequence’ track shows the B73 reference sequence (v3.25) and amino acids from six possible reading frames. The ‘GFF3’ track contains gene structure annotation and expression comparison description of the reference genome. The SNP information including ID, allele frequency and alleles for each individual in the population were listed below in the ‘VCF’ track.

### Variation search

In our database, each SNP or indel is labeled with a unique identifier (ID, e.g. chr1.s_1234). The first string ‘chr’ represents chromosome and the second string indicates the polymorphic type (‘s’ for SNP, ‘i’ for indel). The subsequent number is the chromosome coordinate of a variation. ‘chr1.s_1234’ means the SNP with the coordinate of 1234 bp at chromosome 1. In this interface, users can fetch the genotypes directly through entering the variation ID (Figure 3C). Also, information on variations can be queried by limiting genomic coordinates of the reference genome or gene identifiers (Figure 3C). Furthermore, variations can be filtered by keywords of variation effect prediction (Figure 3C). The acquired variations would be displayed in the results page within a table and have links to the relevant entry in the genome browser to see detail information (Figure 3D).

**Figure 4:**
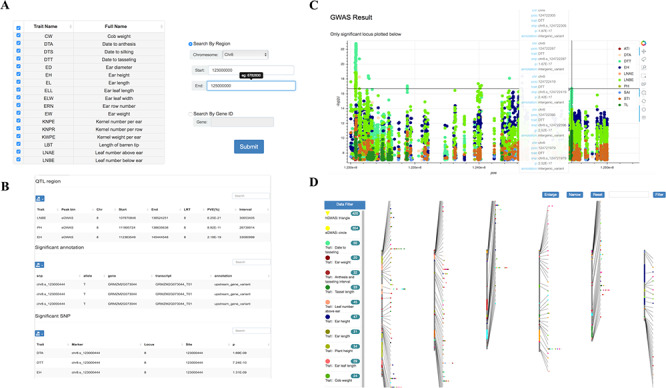
Tools for GWAS results search and visualization in MaizeCUBIC. (**A**) The GWAS signals could be searched by traits or gene ID and variant locations in GWAS search tools. (**B**) And detailed information of significant SNPs for you interested traits will show in downloadable tables on the results pages. (**C**) Each record in the search can be interactively visualizing. And clicking on your interested variations links to the relevant entry in genome browser tool. (**D**) Schematic of the GWAS diagram tool in MaizeCUBIC.

### Gene expression search

A subset of 391 progeny was randomly selected from the CUBIC population for RNA-sequencing, and the gene expression quantification and eQTL mapping results were collected. The expression data with Reads Per Kilobase per Million mapped reads (RPKM) normalization can be searched and downloaded when a list of samples and gene symbols provided through this tool. Moreover, the RPKM-normalized gene expression data and the eQTL mapping results for the 391 samples are also provided on the DOWNLOAD page.

### GWAS diagram

Single-variant-based GWAS and haplotype-based GWAS were used for dissecting genetic mechanisms of over 23 agronomic and yield traits in the population (16). We integrated these GWAS results for multiple traits obtained by diverse methods and dynamic showing their distribution characteristics over the whole chromosome range (Figure 4D). Results can be filtered via tracks on side of the page. The trait names and detailed information display when hovering over each point. And clicking on it links to the relevant entry in the GWAS search tool.

### GWAS search

Detailed information of significant SNPs (*P* < 2.79E-8) for user’s interested traits can be queried by limiting genomic coordinate or gene ID (Figure 4A and B). Also, GWAS Manhattan plot of these SNPs can be interactively visualizing based on a ‘bokeh’ python packages and clicking on given variation links to the relevant entry in genome browser tool (Figure 4C).

### BLASTN/Primer-BLASTN

The BLASTN program was provided to find regions of similarity between nucleotide query to sequences in our local databases and calculates the statistical significance. If the query sequence is less than 50 bp, please choose Primer-BLASTN. Multiple parameters can be adjusted according to demands. Here, we use the BLAST (2) as a backend engine. The results are given in a table format showing the hits found, sequence identifiers for the hits with scoring related data, as well as alignments for the sequence of interest and the hits received with corresponding BLAST scores for the query sequence.

### Primer design

A variety of primer design ways were provided with the software Primer3 (24) as a backend engine. Users can directly input a nucleotide sequence and pick PCR primers in the ‘General Primer Design’ option or choose one of the provided databases and input a genomic region to pick PCR primers to validate genomic sequence or develop molecular markers in ‘Primer Design by Region’ option. Users also can input a variation ID and select upstream and downstream regions to pick PCR primers to validate the variant through the ‘Primer Design by Region Variation ID’ tool. And in all three primer design options, the results will be shown in downloadable text files.

## Discussion and Future Directions

MaizeCUBIC database integrates genomic variation and diverse phenotypes of a well-designed maize synthetic population. Importantly, a series of user-friendly visualization tools have been developed to display the variation information, GWAS results and the recombination haplotype map information of the population. We will continue to optimize the existing database facilities as well as embrace more data and tools. Multi-dimensional omics data including metabolomic and ionomic data of the population will become available once being published. Also, the CUBIC population is a valuable genetic and breeding resource, and some other genetic breeding populations have been developed based on the CUBIC. For example, the 1428 CUBIC lines (24 founders and 1404 offspring) have been crossed to another diverse 30 paternal testers, and over 8000 F1 combinations were obtained and phenotyped across the same five environments. The CUBIC-based F1 hybrid data provide large-scale resources for exploring the genetic of heterosis and facilitate the agricultural data-driven plant breeding design via state-of-the-art technologies such as genomic selection, genome editing and machine learning. All these data will be integrated into this database in the near future. Therefore, the MaizeCUBIC will continuously provide valuable resources for maize community as well as researchers in many other fields.

## Author Contributions

Jianxiao Liu and Jianbing Yan designed and supervised this study. Jingyun Luo, Haijun Liu, Yingjie Xiao and Xiaqing Wang collected data and performed most of the data analysis. Jingyun Luo, Chengcheng Wei and Shikun Cheng worked on the database construction. Jingyun Luo, Jianbing Yan and Jianxiao Liu wrote the manuscript. All authors critically read and approved the manuscript.

## Declaration of Interests

The authors declare no competing financial interests.

**Database URL:** http://cubicmaize.hzau.edu.cn

## Supplementary Material

Paper_baaa044
